# Supplementary figures and images for: Reliable RNA-seq analysis from FFPE specimens as a means to accelerate cancer-related health disparities research
Source: PLoS One. 2025 Apr 21;20(4):e0321631. doi: 10.1371/journal.pone.0321631 (PMC12011225; doi:10.1371/journal.pone.0321631)

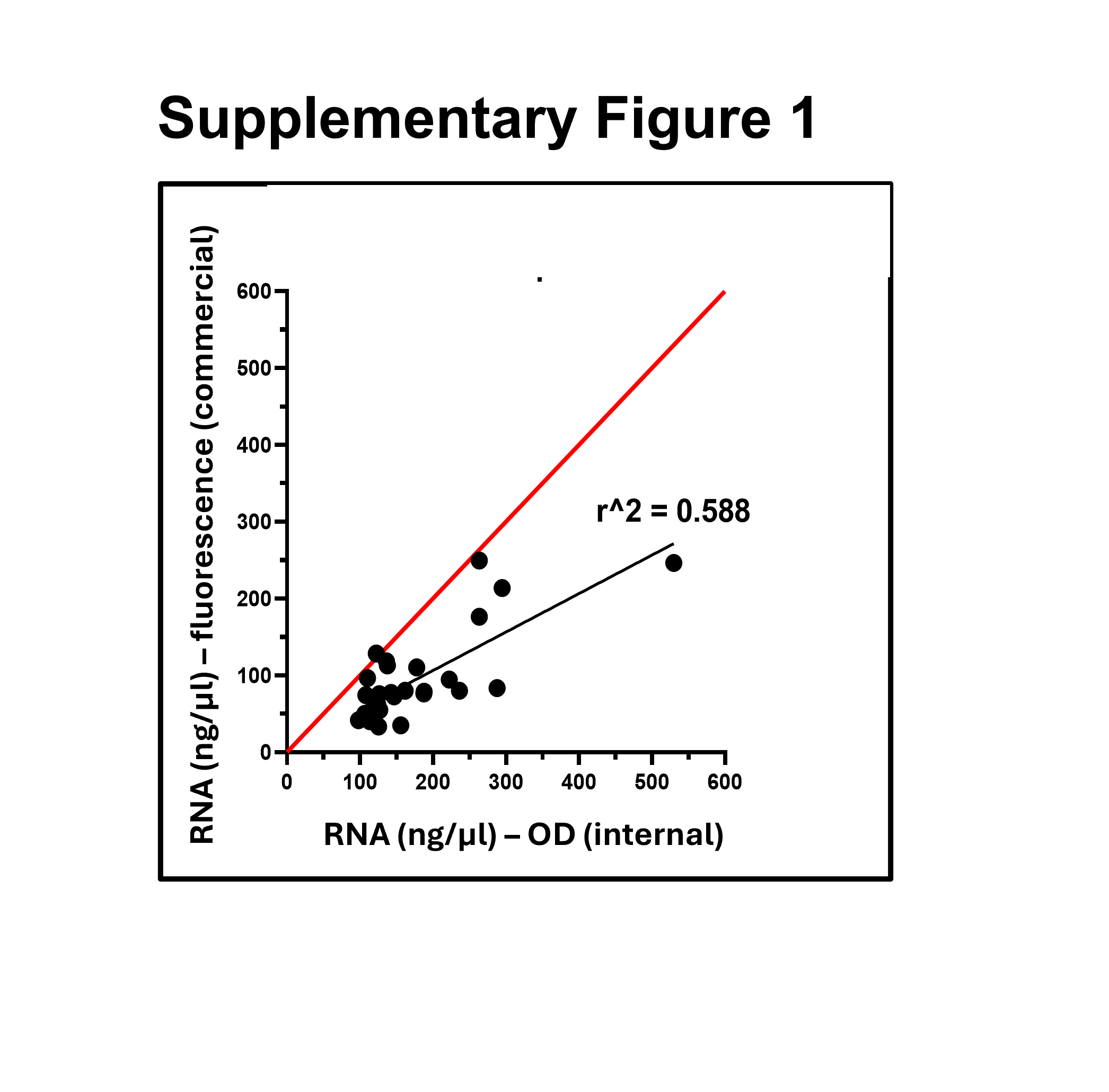

Supplement: S1 Fig — RNA concentrations measured via fluorescence compared to internal laboratory measures using optical density (OD). (TIF) [file pone.0321631.s001.tif]

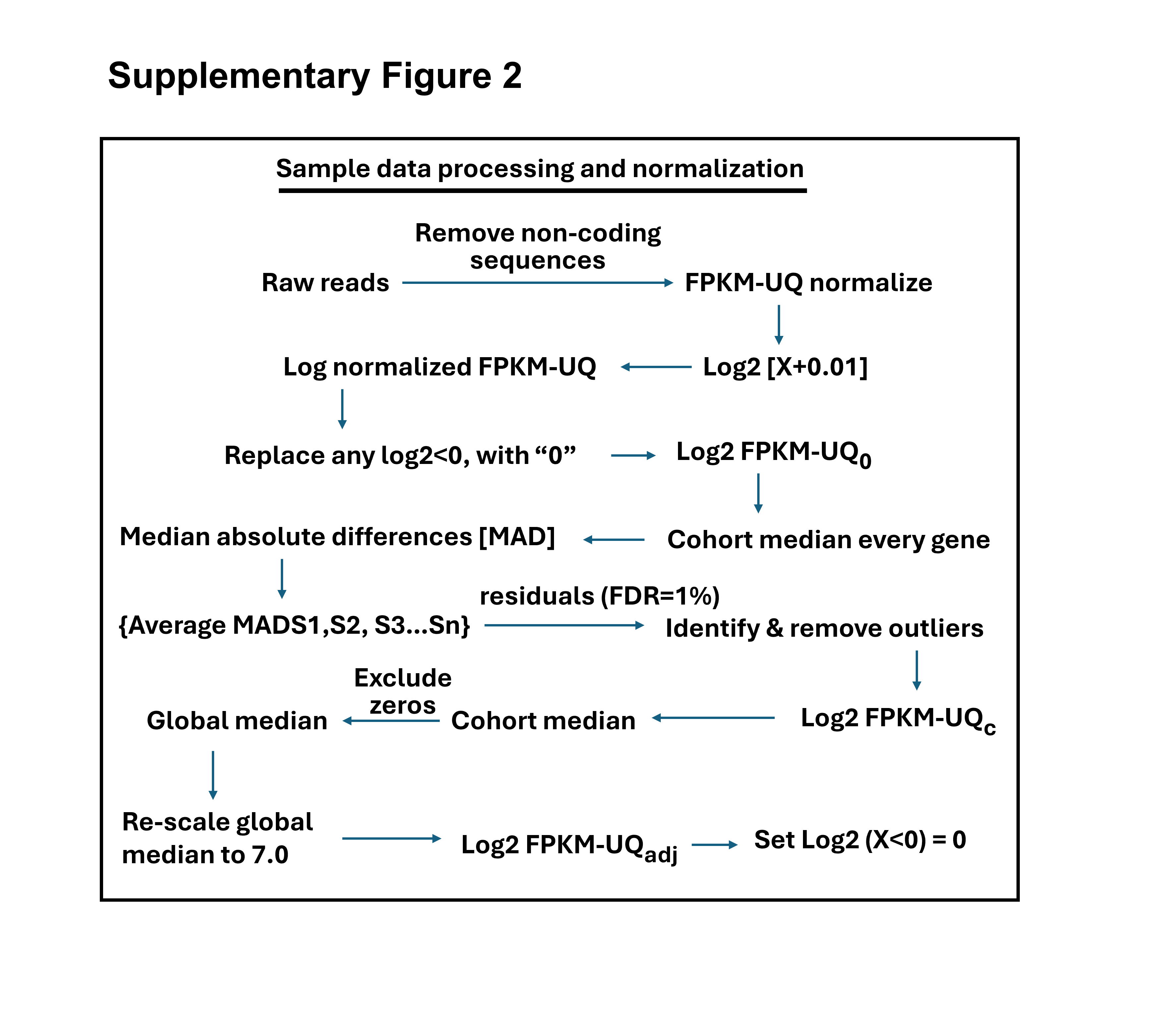

Supplement: S2 Fig — Pipeline for analysis of RNA-seq data inclusive of normalization. (TIF) [file pone.0321631.s002.tif]

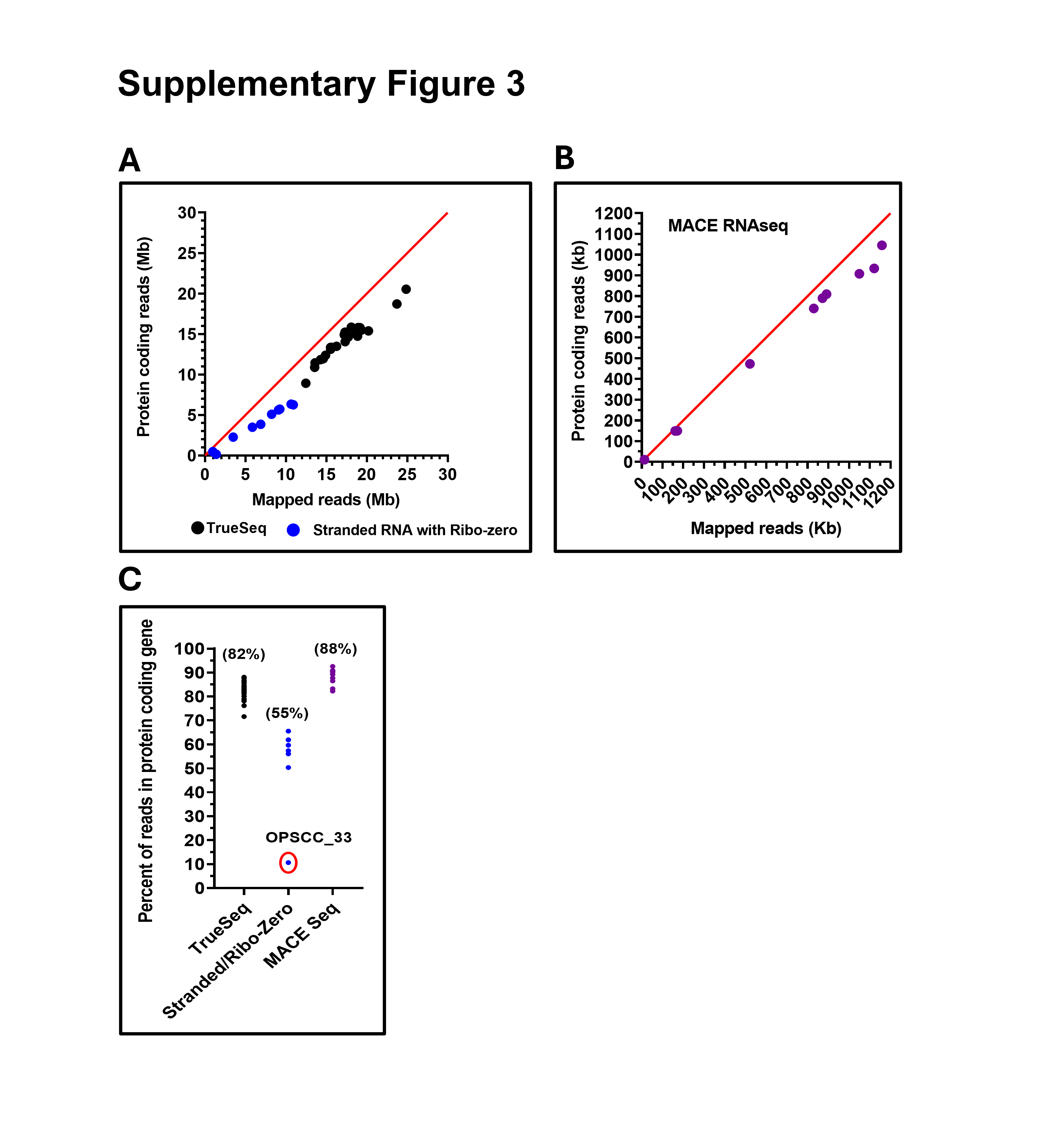

Supplement: S3 Fig — Quantification of coding vs non-coding reads mapped using the True-Seq and Stranded RNA with Ribo-zero platforms (A) and the MACE RNAseq platform (B). Head to head comparison across platforms (C). Sample OPSCC_33, which had the lowest % of protein coding reads (red circle) was also a technical outlier (Supplementary Table 5 in S1 Table). (TIF) [file pone.0321631.s003.tif]

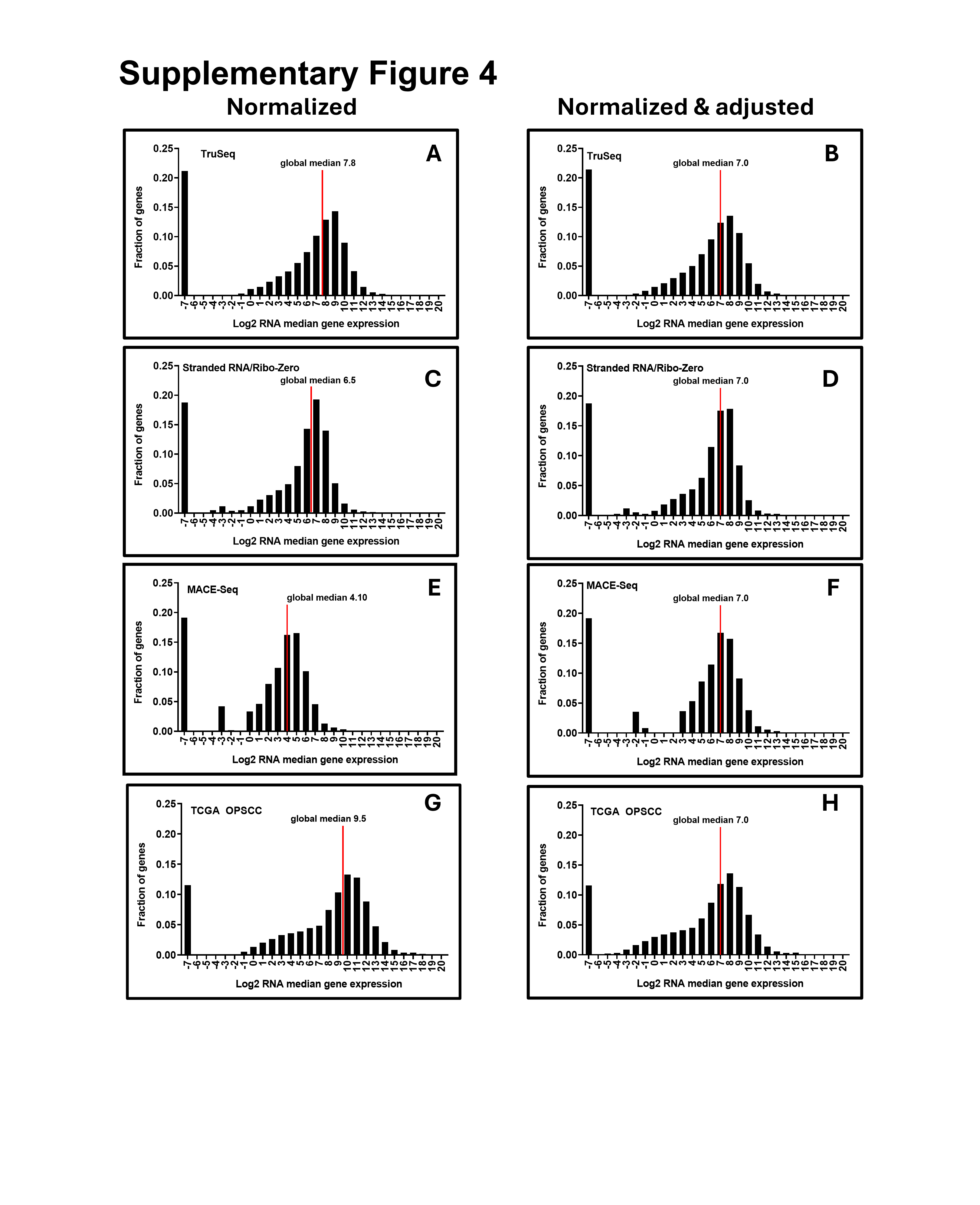

Supplement: S4 Fig — The distributions of gene expression (i.e., median UQ normalized log 2 values) for each platform/cohort, along with the TCGA OPSCC RNA-Seq dataset similarly normalized (A,C,E,G) before global rescaling. Histograms for the rescaled cohorts were also generated (B,D,F,H). (TIF) [file pone.0321631.s004.tif]

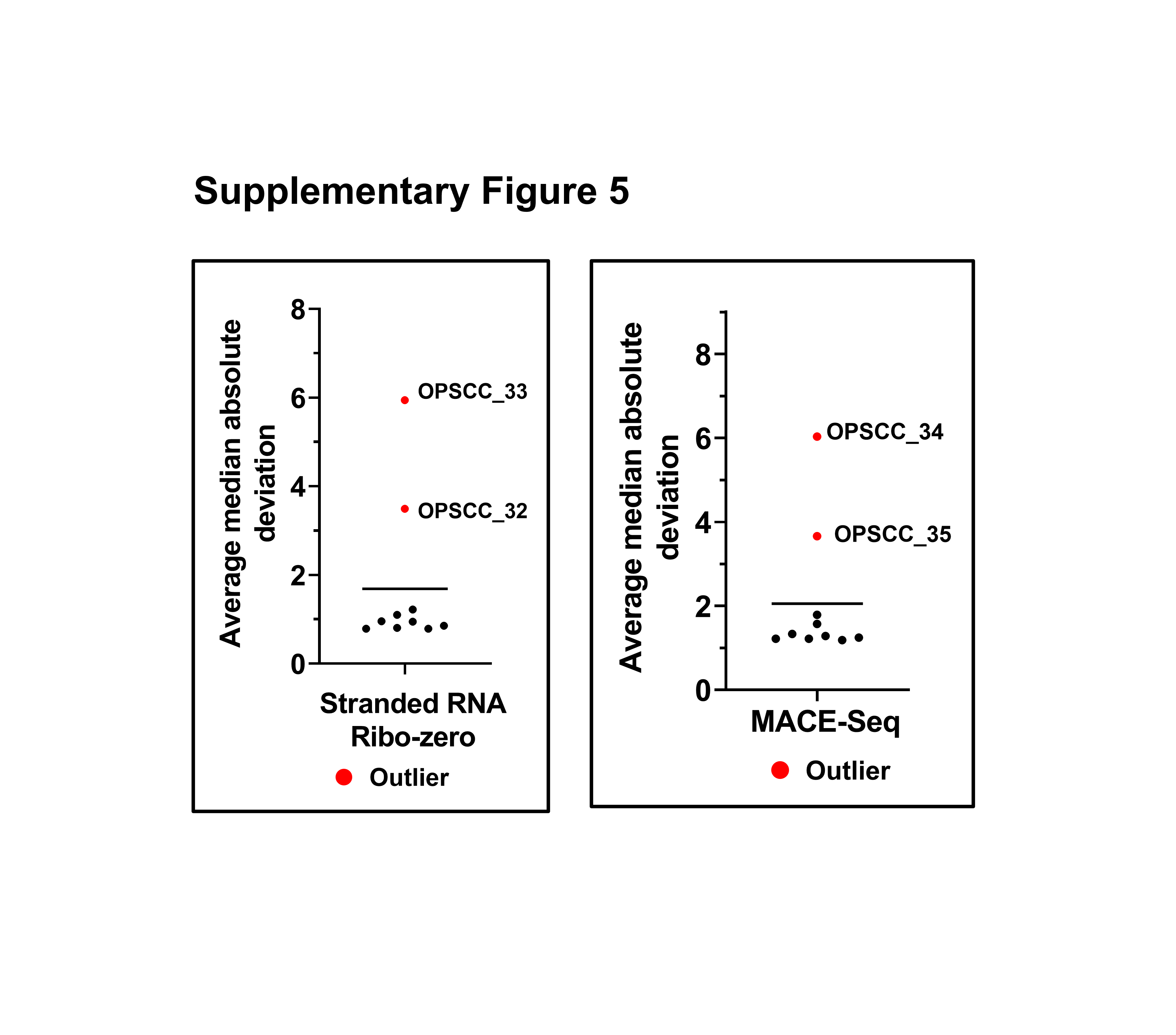

Supplement: S5 Fig — MAD values were averaged across all genes to calculate a sample specific average MAD value. Samples OPSCC_32 and 33 (Stranded RNA/Ribo-zero), OPSCC_34 and 35 (MACE-Seq) were identified as outliers with significantly different MAD values (Supplementary Table 5 in S1 Table). (TIF) [file pone.0321631.s005.tif]

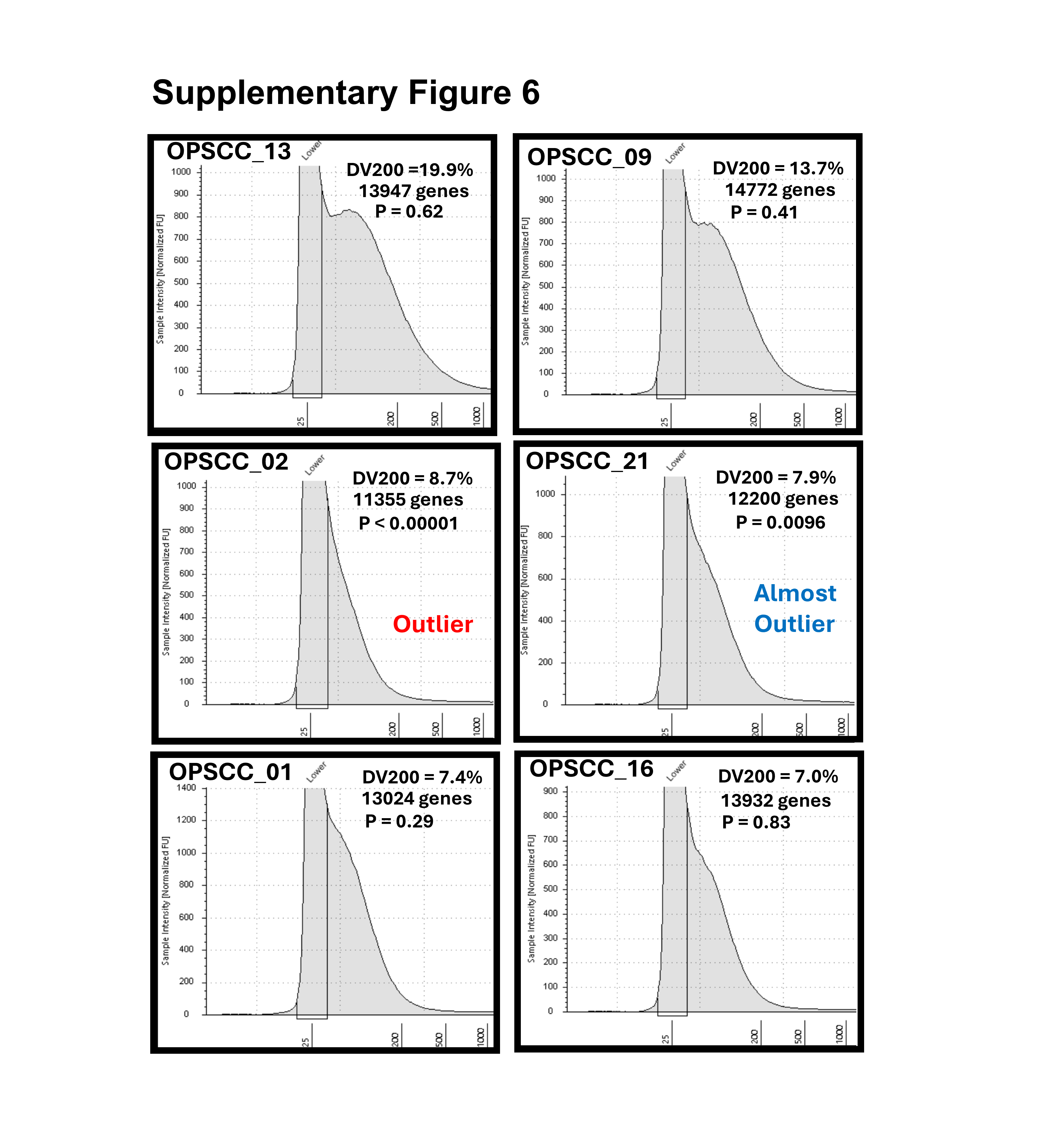

Supplement: S6 Fig — DV200 values and RNA fragment size are shown for representative samples along with the relative number of usable genes and associated p-values from outlier analysis (Supplementary Table 5 in S1 Table). (TIF) [file pone.0321631.s006.tif]

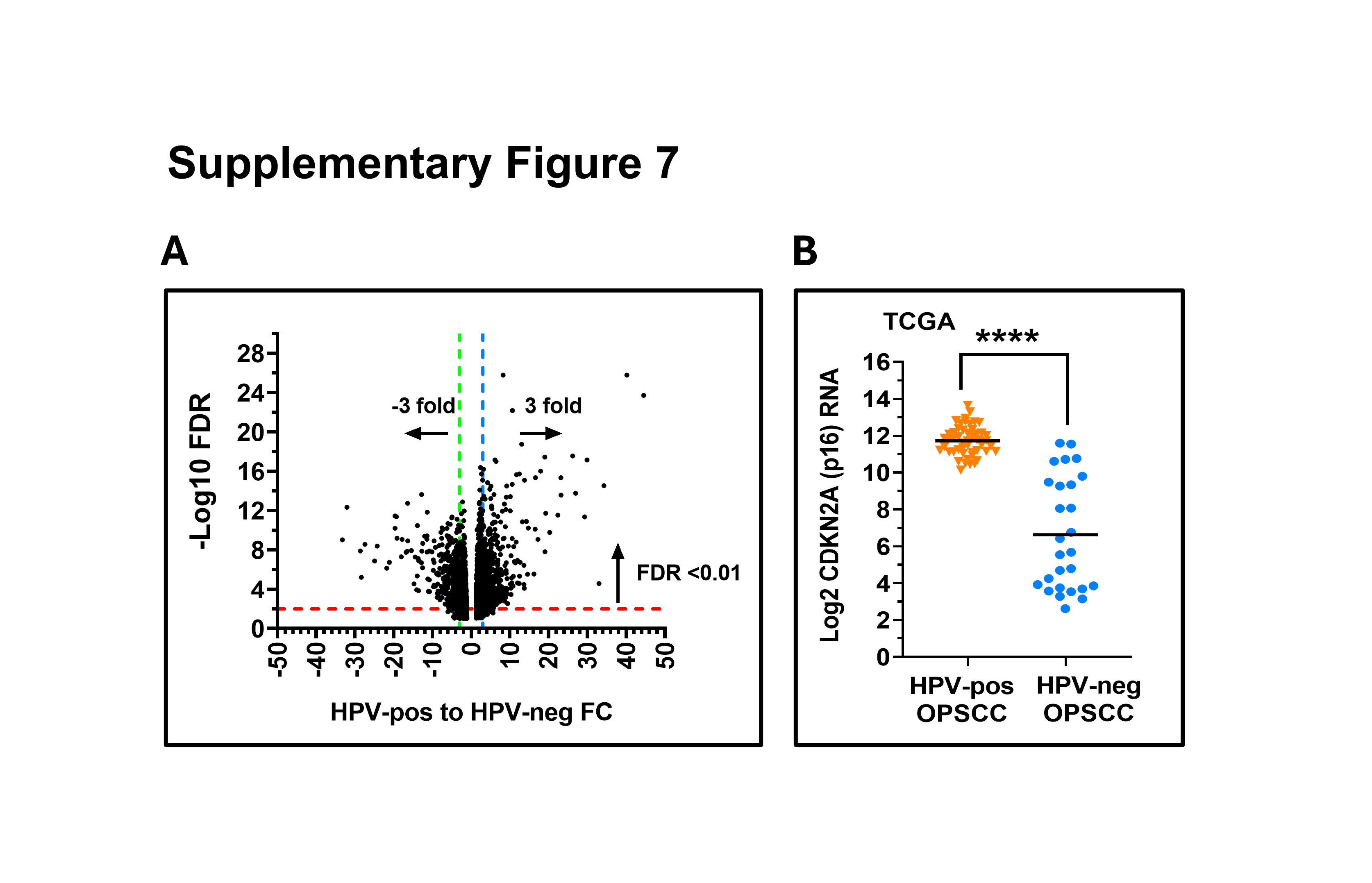

Supplement: S7 Fig — A) Genes that showed ≥3-fold significant (FDR < 0.1) difference up or downregulation based on HPV status in the OPSCC TCGA cohort were identified. B) Confirmation that CDKN2A expression is highly upregulated in HPV-associated (HPV-pos) TCGA samples. ****P < 0.0001. (TIF) [file pone.0321631.s007.tif]

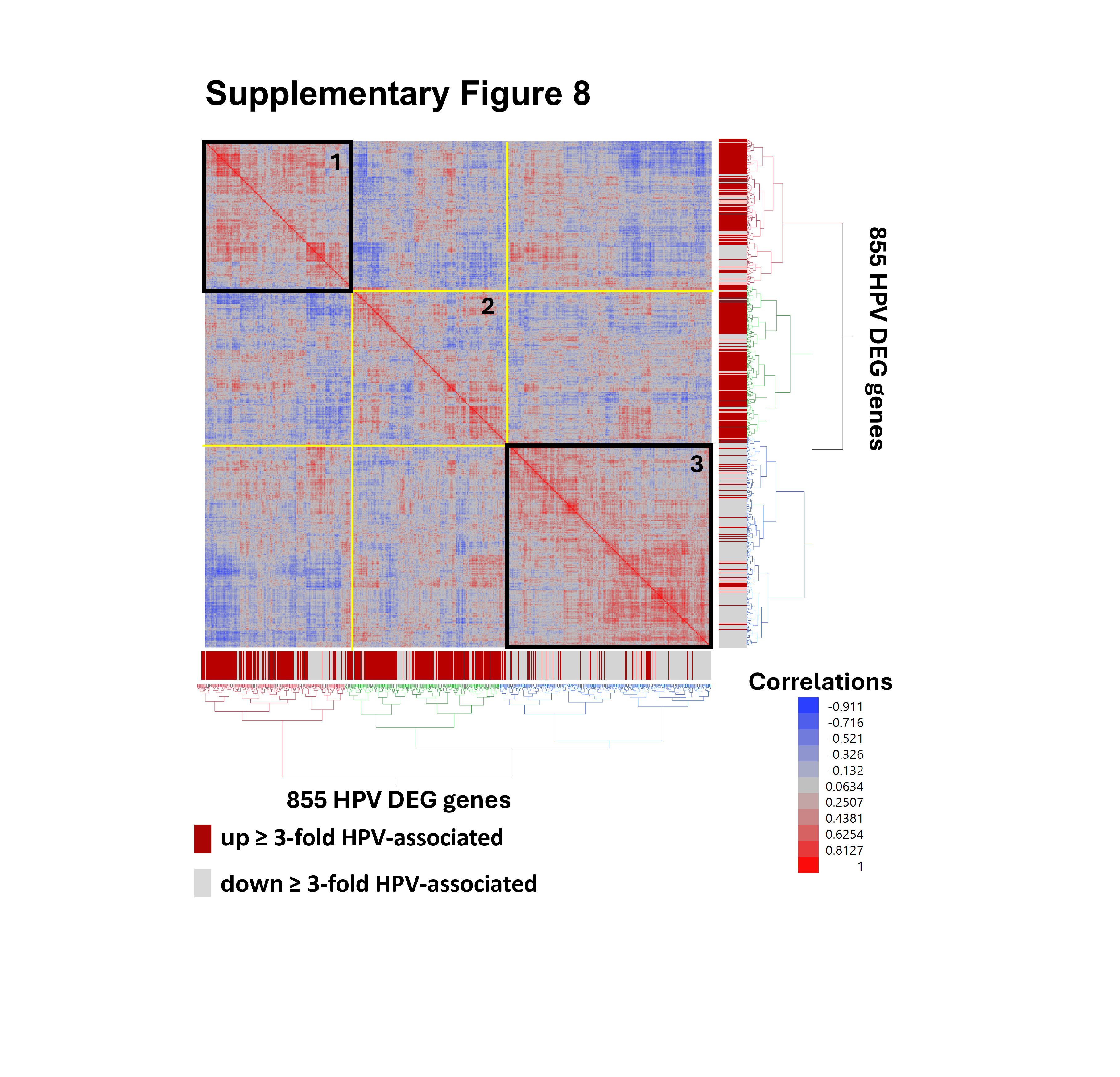

Supplement: S8 Fig — Cross-correlation coefficients of gene expression values within the TruSeq cohort, using the list of 855 DEGs previously associated with HPV status in the TCGA OPSCC samples, were used for unsupervised clustering to identify modules of genes (black boxes) that behaved similarly. Gene clusters 1 and 3 behaved robustly. Genes are annotated vertically and horizontally by whether they were upregulated (red boxes) or downregulated (grey boxes) in the original TCGA cohort according to HPV status. (TIF) [file pone.0321631.s008.tif]

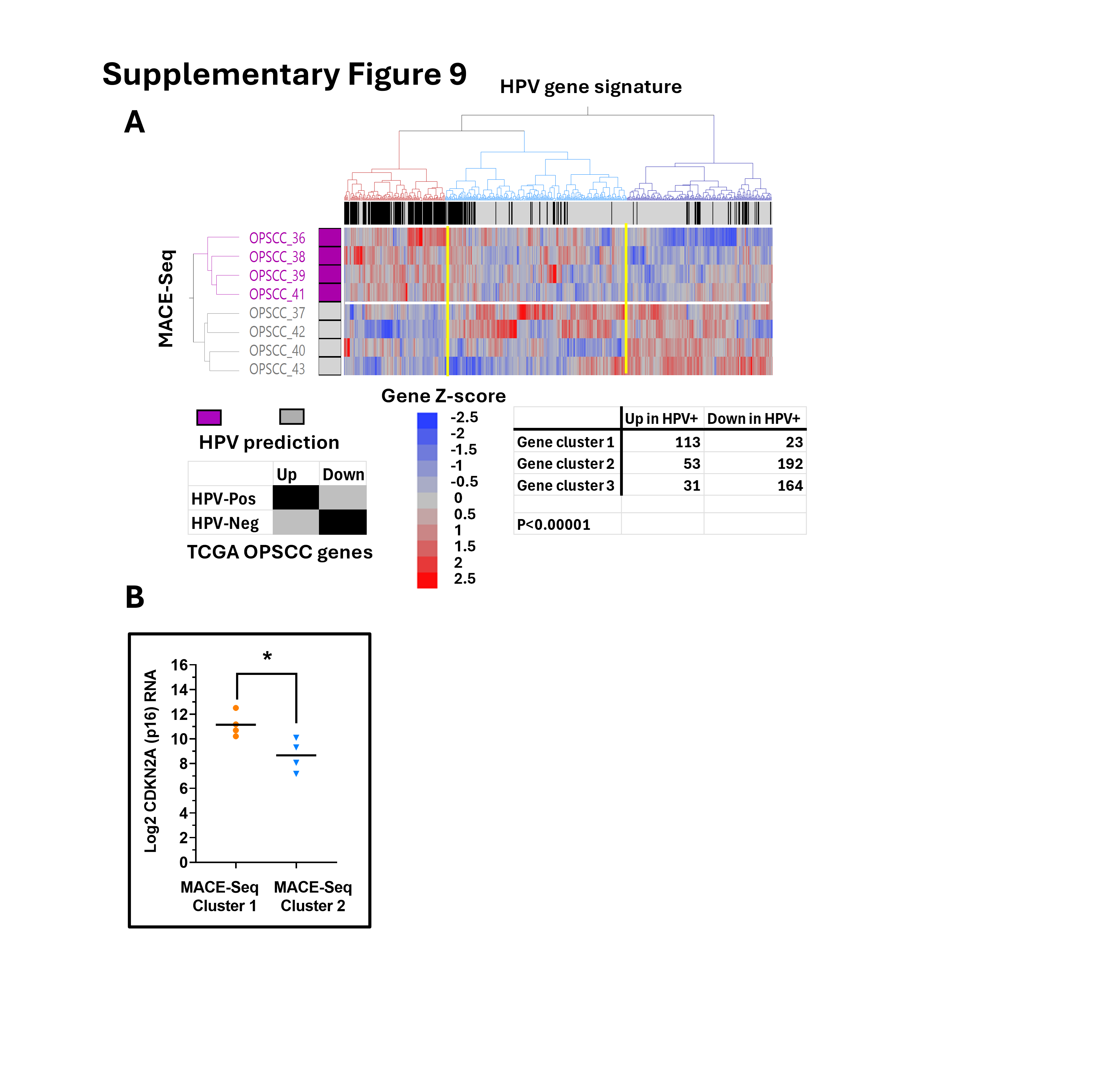

Supplement: S9 Fig — Expression of HPV-associated DEGs (identified from the TCGA OPSCC reference cohort) was used for unsupervised 2-way clustering of MACE-Seq cohort samples (i.e., Ward’s agglomerative hierarchical clustering) to predict HPV status (A). Samples predicted to be HPV-associated (i.e., HPV-Pos) are annotated with purple boxes. The regulation status of genes is annotated across the top of the heatmap with black boxes if they were also upregulated in HPV-associated TCGA samples or grey boxes if they were upregulated in HPV-independent (i.e., HPV-Neg) TCGA samples. Significant enrichment of genes upregulated in TCGA HPV-associated cancers was found in gene cluster 1 (red cluster) and enrichment of genes upregulated in HPV-independent TCGA samples is found in gene cluster 3 (dark blue cluster), demonstrating significant separation (e.g., P < 0.00001 by Chi-square testing). Specimens from sample cluster 1 (purple) predicted to be HPV-associated based on their gene expression pattern had higher CDKN2A (p16) expression than specimens from sample cluster 2 (grey) predicted to be HPV-independent (B). * P < 0.05. (TIF) [file pone.0321631.s009.tif]

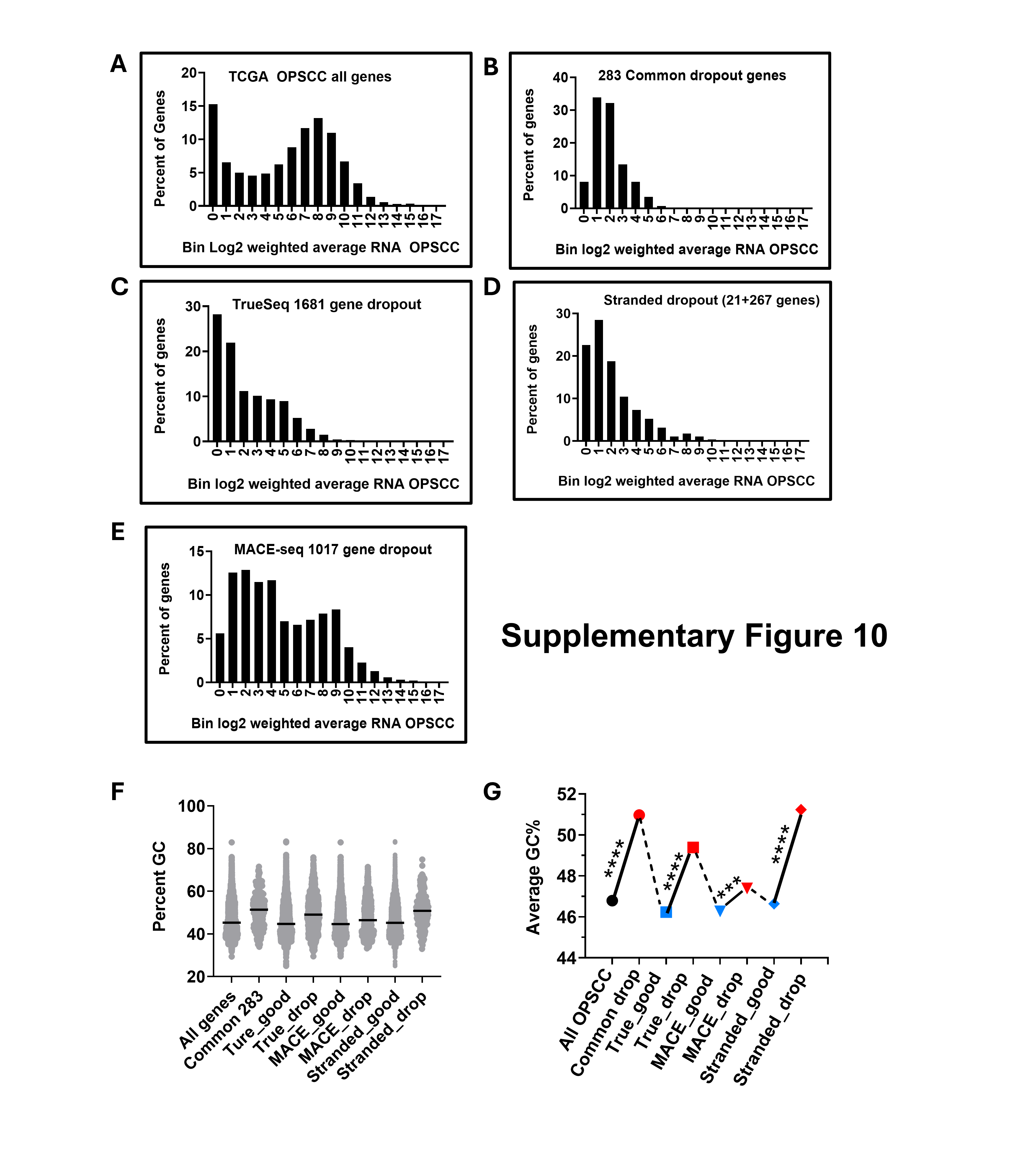

Supplement: S10 Fig — A) Distribution of HPV-associated and HPV-independent weighted averages of RNA expression for all genes using the TCGA OPSCC cohort. B) Distribution of average expression from the TCGA OPSCC cohort for the 283 common dropout genes across all FFPE library platforms. C) Distribution of average expression from the TCGA OPSCC cohort for the 1681 genes that uniquely dropped out from the TruSeq platform. D) Distribution of average expression from the TCGA OPSCC cohort for the 1017 genes that uniquely dropped out from the MACE-Seq platform. E) Distribution of average expression from the TCGA OPSCC cohort for the 248 genes (21 + 267) that uniquely dropped out from the Stranded and/or TruSeq platforms. F) Distribution of GC content among genes that were covered well or dropped out for each platform. G) Statistical comparisons between genes that dropped out and those well covered for each of the platforms or the common 283 dropout genes. The P-values (**** P < 0.00001, *** P < 0.0005) were derived from individual comparisons of averages connected by solid lines after a Tukey’s multiple comparison test. (TIF) [file pone.0321631.s010.tif]

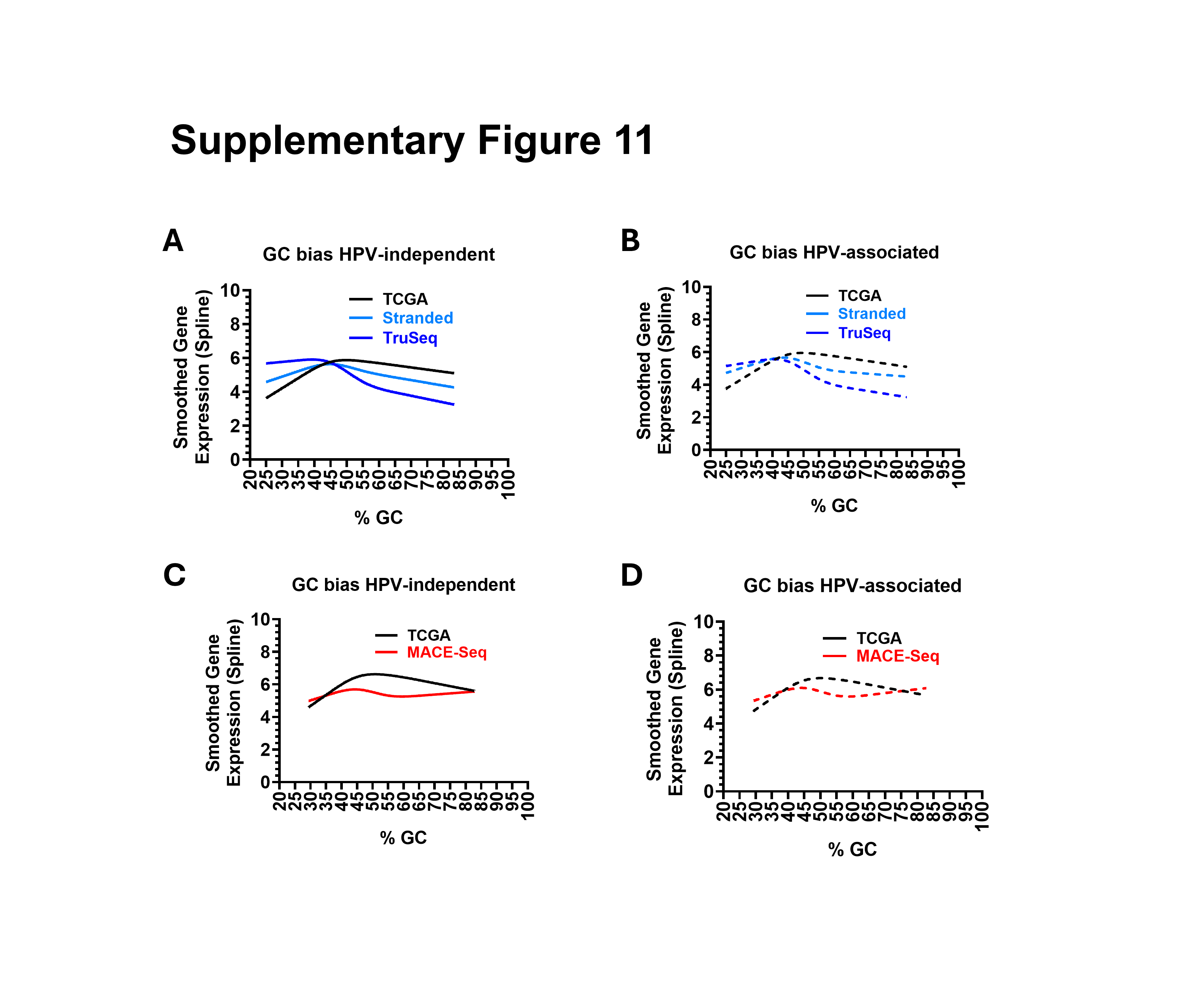

Supplement: S11 Fig — A) Smoothed spline plots comparing average gene expression verses % GC content of canonical transcripts for HPV-independent FFPE samples prepared using the TruSeq (dark blue line) or Stranded (light blue line) libraries compared to HPV-independent TCGA OPSCC samples (black line). B) Smoothed spline plots comparing average gene expression verses % GC content of canonical transcripts for FFPE samples predicted to be HPV-associated and prepared using the TruSeq (dark blue line) or Stranded (light blue line) libraries compared to HPV-associated TCGA OPSCC samples (black line). C) Smoothed spline plots comparing average gene expression verses % GC content of canonical transcripts for FFPE samples predicted to be HPV-independent and prepared using the MACE-Seq approach (red line) compared to HPV-independent TCGA OPSCC samples (black line). D) Smoothed spline plots comparing average gene expression verses % GC content of canonical transcripts for FFPE samples predicted to be HPV-associated and prepared using the MACE-Seq approach (red line) compared to HPV-associated TCGA OPSCC samples (black line). (TIF) [file pone.0321631.s011.tif]

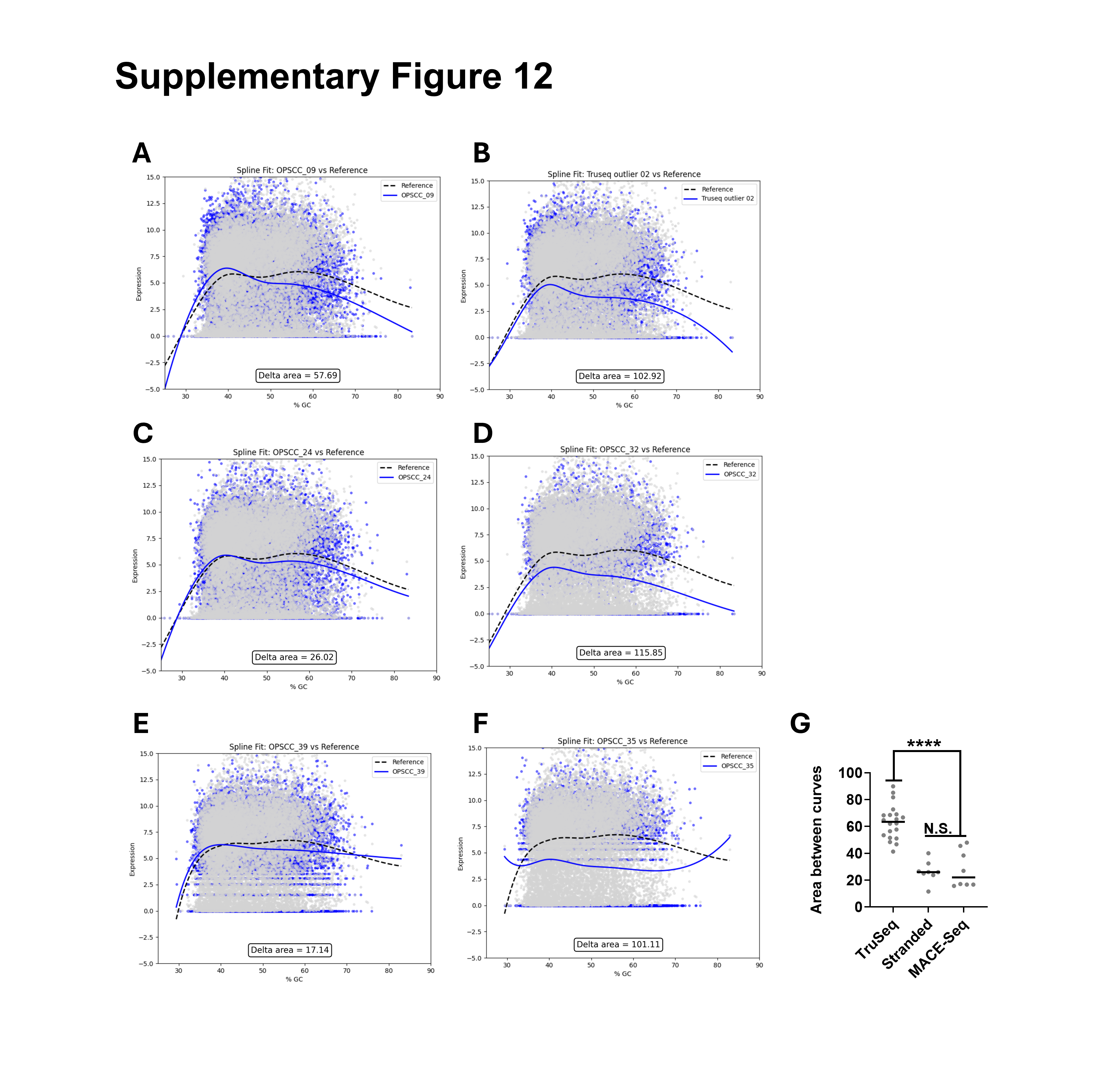

Supplement: S12 Fig — Smoothed spline curves of gene expression verses % GC content of canonical transcripts for individual samples processed using the TruSeq library (A,B), the Stranded library (C,D), or the MACE-Seq library (E,F) compared to a weighted average of HPV-associated and HPV-independent OPSCC TCGA samples for reference. The program computes the area between the reference (OPSCC TCGA samples) and individual sample curves, wherever the sample curve has lower gene expression and displays the value as delta area. Samples identified as technical outliers (B,D, and F) in other steps of the pipeline also show dramatic increases in delta area. G) Scatter plot of average sample delta area values shows significantly more deviation in samples prepared with the TruSeq library compared to those prepared with the other two libraries. **** P < 0.00001. (TIF) [file pone.0321631.s012.tif]
